# Is gravidity associated with COVID-19 vaccination among pregnant women in Jamaica?

**DOI:** 10.1002/rfc2.100

**Published:** 2024-07-04

**Authors:** Jodian A. Pinkney, Laura M. Bogart, Kamali N. Carroll, Lenroy R. Bryan, Givana A. Witter, Dina Ashour, Susanne S. Hoeppner, Rocio M. Hurtado, Ilona T. Goldfarb, Christina Psaros, Emily P. Hyle, Bisola O. Ojikutu

**Affiliations:** 1 Medical Practice Evaluation Center, Department of Medicine, Massachusetts General Hospital, Boston, Massachusetts, USA; 2 Division of Infectious Diseases, Department of Medicine, Massachusetts General Hospital, Boston, Massachusetts, USA; 3 Harvard Medical School, Boston, Massachusetts, USA; 4 RAND Corporation, Santa Monica, California, USA; 5 Charles R. Drew University of Medicine and Science, Los Angeles, California, USA; 6 University of the West Indies, Kingston, Jamaica, West Indies; 7 Department of Psychiatry, Massachusetts General Hospital, Boston, Massachusetts, USA; 8 Division of Maternal Fetal Medicine, Department of Obstetrics and Gynecology, Massachusetts General Hospital, Boston, Massachusetts, USA; 9 Boston Public Health Commission, Boston, Massachusetts, USA; 10 Division of Global Health Equity, Brigham and Women’s Hospital, Boston, Massachusetts, USA

**Keywords:** Caribbean, COVID-19, gravidity, immunization, Jamaica, maternal, pregnant, vaccination

## Abstract

**Introduction:**

In 2021, Jamaica’s maternal mortality ratio doubled as a result of COVID-19-related deaths. Yet, COVID-19 vaccination among pregnant Jamaican women remained low. In the United States, COVID-19 vaccination is lower among pregnant women who have had multiple pregnancies (multigravidas) versus women who were pregnant for the first time (primigravidas). We examined whether this pattern exists in Jamaica.

**Methods:**

A cross-sectional survey of a convenience sample of 79 pregnant Jamaican women recruited from a teaching hospital (May–July 2022) was used to assess self-reported COVID-19 vaccination and medical mistrust beliefs—operationalized as low vaccine confidence, government mistrust, and race-based mistrust—by gravidity. We used modified Poisson regression to estimate adjusted prevalence ratios (aPR) and 95% confidence intervals (CI) for vaccination by gravidity, adjusting for age, education, and comorbidities.

**Results:**

Thirty-nine (49%) of the participants were multigravidas. Socioeconomic status was similar between multigravidas and primigravidas. COVID-19 vaccination was lower in multigravidas (46%) than primigravidas (75%) after adjusting for age, education, and comorbidities (aPR = 0.67, 95% CI = 0.46–0.99; *p* = 0.044). Vaccine confidence was lower in multigravidas (*p* = 0.044). Government mistrust and race-based mistrust did not differ between the two groups.

**Conclusion:**

In Jamaica, multigravidas may have lower COVID-19 vaccine uptake and lower vaccine confidence compared with primigravidas. Understanding the distinct needs of pregnant subpopulations is essential for crafting effective maternal vaccination campaigns.

## INTRODUCTION

In 2021, Jamaica reported a record-high maternal mortality ratio (MMR) of 213 maternal deaths per 100 000 live births, almost half of which were COVID-19-related.^1^ COVID-19 vaccination greatly reduces COVID-19-related morbidity and mortality.^2^ Therefore, identifying subpopulations of pregnant people with disproportionately low vaccine uptake and understanding their unique challenges and needs is important for designing effective vaccination campaigns. In the United States, COVID-19 vaccine uptake was lower for individuals who had been pregnant two or more times (multigravidas) compared with individuals who were pregnant for the first time (primigravidas).^3^ A secondary analysis of vaccine registry data including 2,002 pregnant individuals who delivered in the Mayo Clinic Health System between December 2020 and April 2021 found higher gravidity to be associated with lower COVID-19 vaccination coverage.^3^ Specifically, self-reported COVID-19 vaccination was 49% among primigravida individuals, 24% among second-time pregnant individuals, 21% among third-time pregnant individuals, and 17% among individuals who had been pregnant four or more times.^3^ A similar pattern was observed with maternal influenza vaccine uptake in France.^4^ In Jamaica, COVID-19 vaccines have been widely available across the island free of charge since March 2021.^5^ Despite widespread vaccine availability, a cross-sectional survey of 192 reproductive-aged women in February 2022, found that lower educational level, pregnancy, low vaccine confidence, and government mistrust were associated with low COVID-19 vaccine uptake.^6^ However, it remains unknown whether multi-gravida individuals in Jamaica have lower COVID-19 vaccine uptake compared with primigravida individuals, and if so, the potential reasons for these differences.

## METHODS

We conducted a cross-sectional web-based survey of a convenience sample of pregnant Jamaican women recruited from a teaching hospital (May–July 2022). We examined group differences between multigravida and primigravida women on a set of a priori socio-demographic variables and medical mistrust specific to COVID-19 (with subscales assessing vaccine confidence, government mistrust, and race-based mistrust).^6^ We compared self-reported COVID-19 vaccination and medical mistrust beliefs between primigravida and multigravida women. We used modified Poisson regression to estimate prevalence ratios (PRs) and 95% confidence intervals (CIs) for COVID-19 vaccination, adjusting for age, education level (some college or higher vs. less than college), and medical comorbidities (any medical comorbidity vs. none).

## RESULTS

Table 1 shows baseline study population characteristics by gravidity. Of the 79 pregnant respondents, 39 (49%) were multigravida women. Multigravida women were older than primigravida women (mean [SD] = 31 [4.4] years vs. 28 [5.0] years, respectively, *p* < 0.005). There was no significant difference in the proportion of pregnant women with known socioeconomic predictors of COVID-19 vaccination and healthcare access between the multigravida and primigravida populations in our cohort. Specifically, the proportions of pregnant women who were college-educated, employed, had an income above minimum wage, and had health insurance coverage were not statistically different between the two groups (Table 1). The majority of multigravida and primigravida women had no medical comorbidities (77% and 83%, respectively). Only 4 (10%) multigravida and 3 (8%) primigravida women were in their first trimester of pregnancy. There were no significant differences in trusted sources of COVID-19 vaccine information between the two groups. However, only 5% of multi-gravidas selected social media as a trustworthy source of COVID-19 vaccine information compared with 20% of primigravidas (*p* = 0.087). Self-reported COVID-19 vacci-nation was significantly lower among multigravida women (46%) compared with primigravida women (75%). Multi-gravida women were 33% less likely than primigravida women to report COVID-19 vaccination after adjusting for age, education, and medical comorbidities (aPR = 0.67, 95% CI = 0.45–0.99; *p* = 0.044). Regarding medical mistrust beliefs, multigravida women had lower vaccine confidence compared with primigravida women; government mistrust and race-based mistrust did not differ between the two groups (Table 1).

## DISCUSSION

We found that self-reported COVID-19 vaccination uptake was significantly lower among multigravida women compared with primigravida women after adjusting for age, education level, and medical comorbidities. These results are similar to those from observational vaccination uptake studies in the United States and France.^3,4^ In the French study, it was suggested that the disparity in influenza vaccination could be attributed to multiparous women being less likely to receive a healthcare provider recommendation for vaccination than nulliparous women.^4^ Differences in healthcare provider recommendation for vaccination have important implications because pregnant individuals who receive a healthcare provider recommendation for vaccination are five times more likely to be vaccinated against COVID-19 compared to pregnant individuals who did not receive such guidance.^7^ This could be because healthcare provider recommendation for vaccination plays a crucial role in fostering vaccine confidence, which is a significant determinant of vaccine-related behaviours in the Jamaican pregnant population.^6^

Differences in prenatal education class attendance could be another contributor to disparities in vaccine uptake between multigravida and primigravida women.^8^ Prenatal education classes and prenatal visits/appointments present an opportunity for healthcare providers to discuss maternal vaccines with pregnant individuals and provide their recommendations. However, an observational US-based study found that while 86% of primigravida women attended prenatal education classes, only 14% of multi-gravida women attended.^8^ Greater childcare responsibilities among multigravida women could perpetuate low prenatal education class attendance with one small observational study in Texas reporting lack of childcare as the leading cause for missed healthcare appointments among women of reproductive age.^9^

Similar to an Indian study of 135 pregnant women (73 multigravida, 62 primigravida), our study found no significant difference in socioeconomic status between multigravida and primigravida pregnant women, likely because the study was done at a relatively resource-rich tertiary care hospital.^10^ This finding is important to examine in resource-poor settings, given the significant influence that socioeconomic status has on access to healthcare and COVID-19 vaccine uptake in the general population.

Future mixed-methods research should focus on examining the relationships between gravidity, healthcare provider vaccination recommendations, historical experiences, cultural beliefs, prenatal education class attendance, prenatal visit attendance, vaccine confidence, and vaccine uptake. Understanding these relationships, as well as the unique challenges faced by multigravida women—including how childcare responsibilities may impact their ability to attend prenatal education classes—is key to mapping the vaccine decision-making process within the pregnant population.

## LIMITATIONS

The sample size was small, which may have limited the ability of the final regression model to detect differences in socioeconomic and cultural factors between multigravidas and primigravidas. The study was conducted at a single resource-rich institution in Jamaica, limiting generalizability to resource-poor settings. All responses including comorbidities, gravidity, and receipt of COVID-19 vaccination were self-reported and therefore subject to recall bias. Information on religion, the local vaccine information environment, pregnancy loss, region of residence, residential address, prenatal clinic attendance, and prenatal education was not collected. These factors may differ between multigravida and primigravida pregnant women and could have a differential impact on COVID-19 vaccine uptake.

## CONCLUSION

In Jamaica, multigravida women may have lower COVID-19 vaccine uptake and lower vaccine confidence compared with primigravida women. Understanding the distinct needs of pregnant subpopulations is essential for crafting effective maternal vaccination campaigns and ultimately improving maternal health outcomes overall.

## Supplementary Material

Supplemental 1

Supplemental 2

## Figures and Tables

**TABLE 1:** Population characteristics by gravidity.

	All pregnant women	Primigravida women	Multigravida women	
	(*n* = 79)	(*n* = 40)	(*n* = 39)	
Characteristic	*n* (%)	*n* (%)	*n* (%)	*p* Value^a^
Age, years, mean (SD)	29 (4.9)	28 (5.0)	31 (4.4)	0.005
Race				0.340
Black	76 (97.4)	40 (100.0)	36 (94.7)	
White	1 (1.3)	0 (0.0)	1 (2.6)	
Other	1 (1.3)	0 (0.0)	1 (2.6)	
First trimester of pregnancy	7 (8.9)	3 (7.5)	4 (10.3)	0.155
Health insurance coverage				
Yes	55 (71.4)	31 (79.5)	24 (63.2)	0.113
Employed				
Yes	67 (85.9)	33 (82.5)	34 (89.5)	0.376
Occupation				1.000
Healthcare	9 (13.9)	5 (15.2)	4 (12.5)	
Other	56 (86.2)	28 (84.9)	28 (87.5)	
Education				0.105
Less than college	24 (30.8)	9 (22.5)	15 (39.5)	
Some college or higher	54 (69.2)	31 (77.5)	23 (60.5)	
Income^b^				0.312
Below minimum wage	1 (1.3)	0 (0.0)	1 (2.5)	
Above minimum wage	49 (62.0)	25 (62.5)	24 (61.5)	
Missing	29 (36.7)	15 (37.5)	14 (36.0)	
Comorbidities				
None	63 (79.8)	33 (82.5)	30 (76.9)	0.538
Diabetes	0 (0)	0 (0.0)	0 (0.0)	1.000
Hypertension	3 (3.8)	1 (2.5)	2 (5.1)	0.615
Obesity	4 (5.1)	2 (5.0)	2 (5.1)	1.000
Autoimmune disease	4 (5.1)	2 (5.0)	2 ((5.1)	1.000
Other	5 (6.3)	2 (5.0)	3 (7.7)	0.675
Prior COVID-19 infection				
Yes	20 (25.6)	13 (32.5)	7 (18.4)	0.155
Perceived risk of getting COVID-19 infection in the coming month, *M* (SD)	−0.9 (0.9)	−1.0 (0.9)	−0.8 (0.9)	0.438
Sources of COVID-19 information				
Healthcare providers (e.g., doctors or nurses)	51 (64.6)	24 (60.0)	27 (69.2)	0.391
Local public health officials	26 (32.9)	10 (25.0)	16 (41.0)	0.130
News (TV)	26 (32.9)	15 (37.5)	11 (28.2)	0.379
News (Internet)	26 (32.9)	16 (40.0)	10 (25.6)	0.175
Federal government health agencies (e.g., CDC^c^ or Ministry of Health)	23 (29.1)	15 (37.5)	8 (20.5)	0.097
People I know (e.g., friends, family, neighbours, or co-workers)	18 (22.8)	10 (25.0)	8 (20.5)	0.635
Social media (Facebook, Twitter, WhatsApp)	10 (12.7)	8 (20.0)	2 (5.1)	0.087
Federal government officials (JLP^d^/Holness administration)	4 (5.1)	4 (10.0)	0 (0)	0.116
Self-reported COVID-19 vaccination				
Yes	48 (60.8)	30 (75.0)	18 (46.2)	0.009
Factor subscales				
Low vaccine confidence, *M* (SD)	−0.2 (0.8)	−0.4 (0.8)	0.0 (0.8)	0.044
Government mistrust, *M* (SD)	0.1 (0.8)	0.0 (0.8)	0.3 (0.8)	0.151
Race-based mistrust subscale, *M* (SD)	−0.3 (0.6)	−0.3 (0.7)	−0.3 (0.6)	0.981

a *p* Values were estimated using Chi-square or Fisher exact tests for categorical variables, and Kruskal–Wallis test for continuous variables.

b As of 1 April 2022, Jamaica’s minimum wage increased from JA$7000 (equivalent USD 45) to JA$9000 (equivalent USD 58) per 40-h work week, however for the purposes of this paper we considered < JA$7000 minimum wage based on the approved IRB.

c CDC, United States Centers for Disease Control and Prevention.

d JLP, Jamaica Labor Party (one of two major political parties in Jamaica).

## Data Availability

All data relevant to the study are included in the manuscript. The original anonymous data set can be found in the supplemental material.
